# Optomechanical Design and Application of Solar-Skylight Spectroradiometer

**DOI:** 10.3390/s21113751

**Published:** 2021-05-28

**Authors:** Zhaoyang Qi, Jianyu Li, Wenqing Xu, Wenyue Zhu, Fengying Sun, Yao Huang, Gang Xu, Congming Dai

**Affiliations:** 1 Key Laboratory of Atmospheric Optics, Anhui Institute of Optics and Fine Mechanics, HFIPS, Chinese Academy of Sciences, Hefei 230031, China; Qzy0312@mail.ustc.edu.cn (Z.Q.); wqxu@aiofm.ac.cn (W.X.); zhuwenyue@aiofm.ac.cn (W.Z.); sfy0717@mail.ustc.edu.cn (F.S.); hyhy@aiofm.ac.cn (Y.H.); xiaoxug@mail.ustc.edu.cn (G.X.); cmdai@aiofm.ac.cn (C.D.); 2 University of Science and Technology of China, Hefei 230026, China

**Keywords:** spectroradiometer, atmospheric optics, embedded Linux, sensor networks

## Abstract

Using a solar radiometer is an effective approach for improving the remote sensing of solar irradiance distribution and atmospheric composition. Long-term development of a solar scanning radiometer enables frequent and reliable measurement of atmospheric parameters such as the water vapor column and aerosol optical properties. However, the discrete wavelength radiometer has encountered a bottleneck with respect to its insufficient spectral resolution and limited observation waveband, and it has been unable to satisfy the needs of refined and intelligent on-site experiments. This study proposes a solar-skylight spectroradiometer for obtaining visible and near-IR fine spectrum with two types of measurement: direct-sun irradiance and diffuse-sky radiance. The instrument adopts distributed control architecture composed of the ARM-Linux embedded platform and sensor networks. The detailed design of the measuring light-path, two-axis turntable, and master control system will be addressed in this study. To determine all coefficients needed to convert instrument outputs to physical quantities, integrating sphere and Langley extrapolation methods are introduced for diffuse-sky and direct-sun calibration, respectively. Finally, the agreement of experimental results between spectroradiometers and measuring benchmarks (DTF sun-photometer, microwave radiometer, and Combined Atmospheric Radiative Transfer simulation) verifies the feasibility of the spectroradiometer system, and the radiation information of feature wavelengths can be used to retrieve the characteristics of atmospheric optics.

## 1. Introduction

Since solar energy is selectively absorbed and scattered by atmospheric molecules in transmission, the radiation distribution on the ground characterizes many atmospheric optics properties [1]. In particular, visible and near-IR wavelength shortwave radiation is of great significance to theoretical meteorology research, atmospheric detection and validation of satellite aerosol retrievals [2,3]. Hence, it is critically important to understand and describe solar irradiation information [4]. The accurate measurement of shortwave radiation has been the prerequisite for assessing its influence on atmospheric circulation and improving space imaging accuracy in the engineering field [5].

When radiation detection technology is underdeveloped, the ISO9845 can provide an appropriate standard spectral distribution for the scientific research on direct beam radiation extinction [6]. However, the prospect of fully understanding radiation can only be realized with verification and validation by a high-precision solar radiometer. Over the past few decades, radiometers have undergone long-term research to realize atmospheric observation, instrument calibration and data processing [7,8]. The polarized CE318-DP radiometer system used in the China Solar/Sky Radiation Observation Network is a typical example that standardizes regional ground-based water vapor and aerosol observations [9,10]. However, the instruments have to take a few seconds to traverse a filter wheel sequence while performing measurements, limiting their time resolution. In contrast to the CE318 radiometer, the multispectral sun-photometer (SSARA) manufactured at the Meteorological Institute Munich can measure the radiation of all channels simultaneously [11]. Owing to the design of their electronics, the channel amplifiers have a high time constant during scanning. In addition, the instrument has not overcome the restraint of limited measurement channels. The “Fengyun” meteorological satellites of aerospace engineering provide support for the development of spectroradiometers with a spectral range of 400~1050 nm, with the prospect to obtain refined solar radiation distributions for meteorological modeling and ecological research [12,13]. A spectroradiometer is an optical precision instrument integrated with electrical and optomechanical technology. Its implementation requires that the master control system has a series of characteristics such as real-time communication, embedded algorithms and data processing. With the developments of microprocessors, system-on-chip and sensor detection, the ARM microprocessor system presents integrated and intelligent application advantages in atmospheric detection and marine environment monitoring [14]. Moreover, previous work [15,16] proved the feasibility of building a radiometer system on the ARM platform to reduce energy consumption and size. Therefore, an improved spectroradiometer system based on embedded ARM-Linux is developed in this study, which simultaneously measures the visible and near-IR spectrum in the whole sky.

The organization structure of this article is as follows: Section 2 introduces the optical path of the radiometer in detail, and analyzes how to introduce the communication protocol family into the master control system to realize distributed control of measurement components. Brief descriptions of the radiometric calibration method are given in Section 3, and Section 4 presents how to retrieve continuous spectral transmittance, water vapor column and other atmospheric parameters according to the radiation information of characteristic wavelengths.

## 2. Instrument Characterization

### 2.1. Overview Design

Wide observation waveband, high spectrum resolution, and flexibility in configuration modes are the basic characteristics of the instrument framework. The solar-skylight spectroradiometer can observe the wideband solar spectrum range of 380~1100 nm, with spectral resolution of 0.1 nm. This instrument integrates two types of sun-photometric measurement: direct-sun irradiance and diffuse-sky radiance. The coordination of a two-axis turntable, measuring probe and embedded control system aids the development of the radiometer. At the same time, peripheral components such as the temperature control system also contribute to the improvement of instrument measurement accuracy.

**Figure 1:**
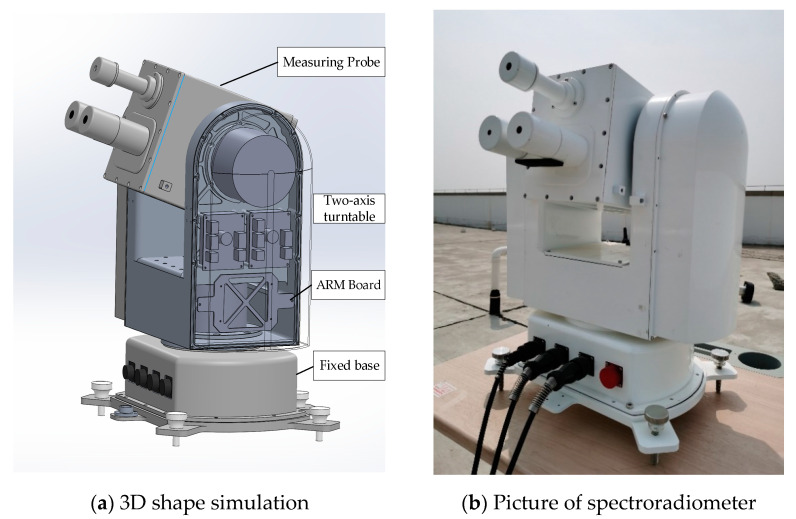
Schematic diagram of instrument.

A schematic diagram of the spectroradiometer is shown in Figure 1. The two-axis turntable adopts a symmetrical U-shaped structure to reduce the moment of inertia, and supports the measuring probe in the inertial operation space. The measuring probe can rotate in two orthogonal dimensions of horizontal axis and pitch axis to realize steady and accurate tracking of the target in the whole sky, while observing the solar spectrum from visible to near-IR. The temperature control technology provides probe cavity with working environment of rapid thermal homogenization, constant temperature control, and heat insulation protection. With the ARM microprocessor as the central microprocessor and Linux OS as the software platform, the embedded system is responsible for task scheduling and real-time communication with radiometer components. Based on the rational configuration of the communication protocol family, the data from sensor networks are integrated and merged under the program control. Table 1 below depicts the performance indicators of the solar-skylight spectroradiometer:

### 2.2. Light-Path Design

**Figure 2:**
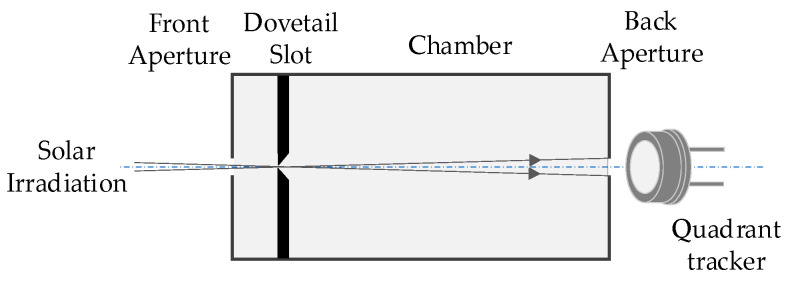
Sun tracking light-path.

Accurate tracking of the sun is the basis for obtaining direct solar irradiance and sky radiance, with the sun as the reference point. Therefore, designing the tracking light-path should satisfy the requirement that the chamber is perpendicular to the solar plane, and the solar spot completely falls on the photosensitive surface of the quadrant detector, as shown in Figure 2. The astronomical sun trajectory algorithm built in the microprocessor calculates the solar position based on time, latitude and longitude, after which the quadrant detector ensures the tracking accuracy. The above dual-mode tracking technology realizes the automatic and precise tracking of the solar center prior to a programmed measurement sequence.

**Figure 3:**
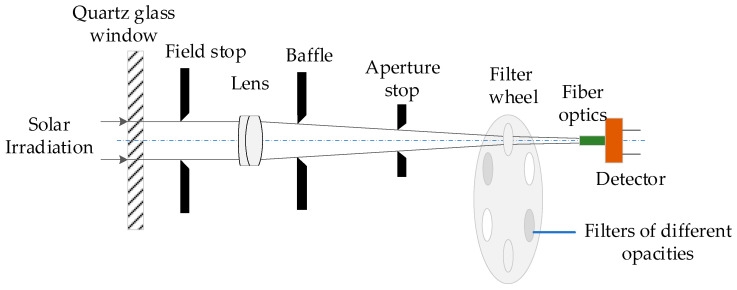
Spectrum acquisition light-path.

Figure 3 sketches the spectrum acquisition light-path used to effectively collect solar irradiation. The light-path is mainly composed of diaphragm groups, a doublet lens, neutral density filters of different opacities, optical fibers and Sony silicon CCD-array detectors. The design of the front-end light-path must satisfy the 32′ solar disc angle, and take into account the manufacture error and adjustment margin of the field of view (FOV). After solar irradiation passes through the dustproof quartz glass window, the field diaphragm is responsible for limiting the luminous flux and FOV. An achromatic doublet lens and evanescent light baffle can effectively realize straylight rejection. The focal length of the doublet lens is 69.8 mm, and the corresponding solar spot size is about 0.65 mm, so the photosensitive surface size of the spectral detector is designed to be more than 1 mm. Moreover, the aperture diaphragm placed in front of the photosensitive surface limits the receiving field of view to 0.8°. The above optical path design can not only allow the solar spot to fall into the receiving surface, but also have enough space margin for adjusting the dovetail groove to reduce the parallelism error between measuring the optical axis and tracking the optical axis [17]. In order to avoid overexposure of the CCD detector when pointing at the sun, neutral density filters with 0.1% or 0.3% transmittance are arranged into a rotating wheel, respectively. As the incident sunlight passes through the filters, the spectrum range of 300~1700 nm is selected and attenuated, after which the optical fibers and fiber couplers couple the solar radiation to the spectrometer. Then, the spectrometer converts the physical quantities to electrical signals and analyzes the spectral composition.

### 2.3. Radiometer System with Embedded Linux

**Figure 4:**
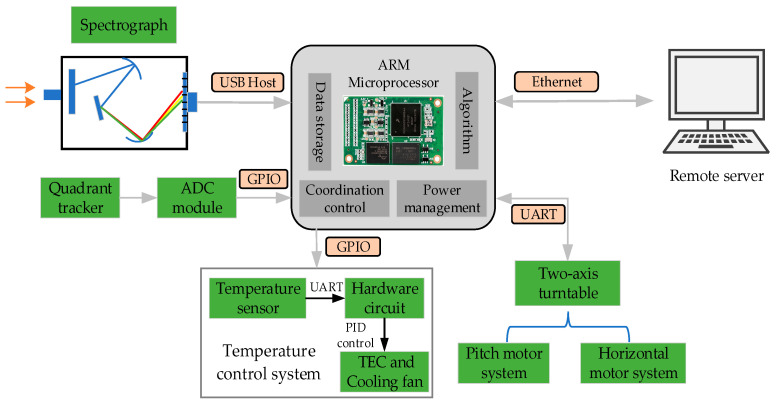
Architecture diagram of embedded platform.

The high-performance ARM Cortex-A7 microprocessor with an embedded Linux system is implemented for the coordination of a two-axis turntable, measuring probe and temperature control module. ARM-Linux architecture can realize minimal energy consumption and support data processing with the referenced algorithms [18], which is an important advantage in long-term observations. With the introduction of the communication protocol family (UART, GPIO, and USB), the embedded system can flexibly regulate the sensor networks to ensure cooperative work. Under the healthy cycle of the control algorithm, spectrum data can be regularly copied to the remote server by means of the TCP/IP network transmission protocol. Figure 4 illustrates the software framework of the radiometer system:

#### 2.3.1. Two-Axis Turntable

**Figure 5:**
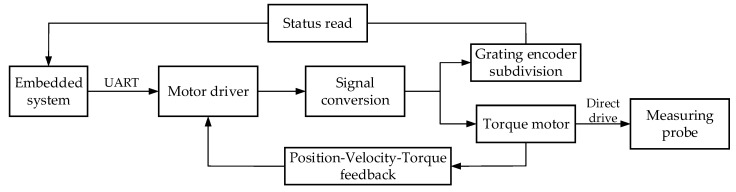
Schematic illustration of turntable control.

The turntable is the carrier of electrical and optical components in the measuring probe; Figure 5 shows the turntable control flow. The serial communication protocol is selected to simplify data transmission and realize reliable communication between the microprocessor and turntable [19]. According to the programmed protocol format, the signals sent by the ARM board serial will traverse the built-in command preset in the driver, after which the driver converts the signals into voltage parameters to adjust the Brushless Direct Torque motor. Based on the hardware foundation consisting of drivers, encoders and gratings, the “position-velocity-torque” force control loop in the software enables the two-axis turntable to have positive transmission stability and positioning accuracy better than 40 arcsec [20]. The direct drive technology of the torque motor frees the transmission device between the motor and turntable load [21], and eliminates the errors caused by the transmission chain (friction vibration, response lag, and elastic deformation). Through the stable operation of the program loop, the microprocessor could convert the displacement deviation into control signals of the turntable, and monitor the state of the turntable in real time.

#### 2.3.2. Measuring Probe

The prominent advantages of the spectroradiometer lies in the spectral spectroscopy and multi-channel array detector technology. While the radiometer scans on the trajectory, the measuring probe mounted on the two-axis turntable will obtain the visible and near-IR spectrum. The fiber optics spectrometer manufactured by Ocean Optics Co. Ltd. (Dunedin, FL, USA) is a precise instrument combined with programmable electronics, CCD-array detector and high-speed transmission. Therefore, by splicing the spectrum of the visible and near-IR spectrometers in the program, the radiometer can quickly measure the spectrum-wide range of 380~1100 nm.

**Figure 6:**
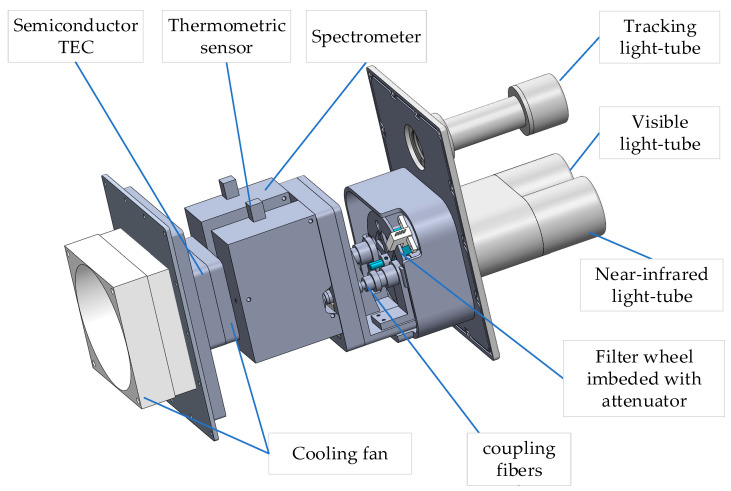
Three-dimensional structure of spectrum probe.

In order to ensure high-speed and large-capacity data transmission, the USB communication protocol is adopted for the cooperative communication between the microprocessor and spectrometer. Owing to the facilities of the open-source Libusb-0.1.9, embedded Linux could operate the USB spectrometer through an efficient driverless method without considering the compatibility of various kernel versions [22], which leads to software simplification. In addition, while the spectrometer collects the solar spectrum, the noise subtraction program and linearity correction program are triggered to eliminate the dark noise and minimize the influence of electric noise. Figure 6 shows the three-dimensional structure of the spectral measuring probe.

#### 2.3.3. Temperature Control System

The potential uncertainty of the spectrum measurement comes from ineliminable electric noise and the temperature dependence of spectral response, and it is necessary to carry out temperature control design of the measuring probe. The temperature control system is mainly composed of a thermometric sensor, semiconductor TEC and hardware circuit [23]. The sensor measures the cavity temperature, after which the microprocessor adjusts the operation of the TEC and air-cooling system based on the built-in PID algorithm. The temperature control system keeps the temperature in the probe cavity constant with maximum offset of 0.5 °C, which improves the accuracy of the spectral measurement.

### 2.4. Workflow

**Figure 7:**
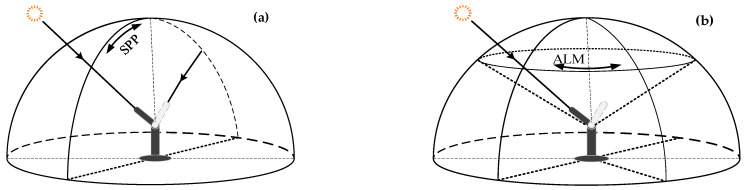
Diffuse-sky observation diagram of spectroradiometer: (**a**) SPP; (**b**) ALM.

The spectroradiometer is designed to perform direct-sun irradiance measurements and diffuse-sky detection; fixed-point observation is part of the latter:

- During the process of direct-sun measurement, solar tracking and radiation collection programs circulate in the microprocessor, so that the direct solar irradiance of the whole day sequence from sunrise to sunset can be obtained. Using the relative flux of directly transmitted radiance varying with the zenith angle, atmospheric parameters such as atmospheric transmittance and water vapor column can be calculated by the inversion model.
- In diffuse-sky measurement, dual-mode tracking technology is executed to accurately track solar position, after which the spectroradiometer scans the solar principal plane (SPP) and almucantar (ALM) shown in Figure 7 at non-equal intervals to obtain the whole sky radiance.

- As for fixed-point observation, the measuring probe aims at a specific direction to obtain continuous fixed-point radiance. The measurement cycle and integration time of the spectrometer can be dynamically adjusted, but the increase in integration time will lead to the elevation of dark noise and compression of the signal-to-noise ratio. Therefore, the adjustment of integration time adopts the principle of 1~320 ms classification.

**Figure 8:**
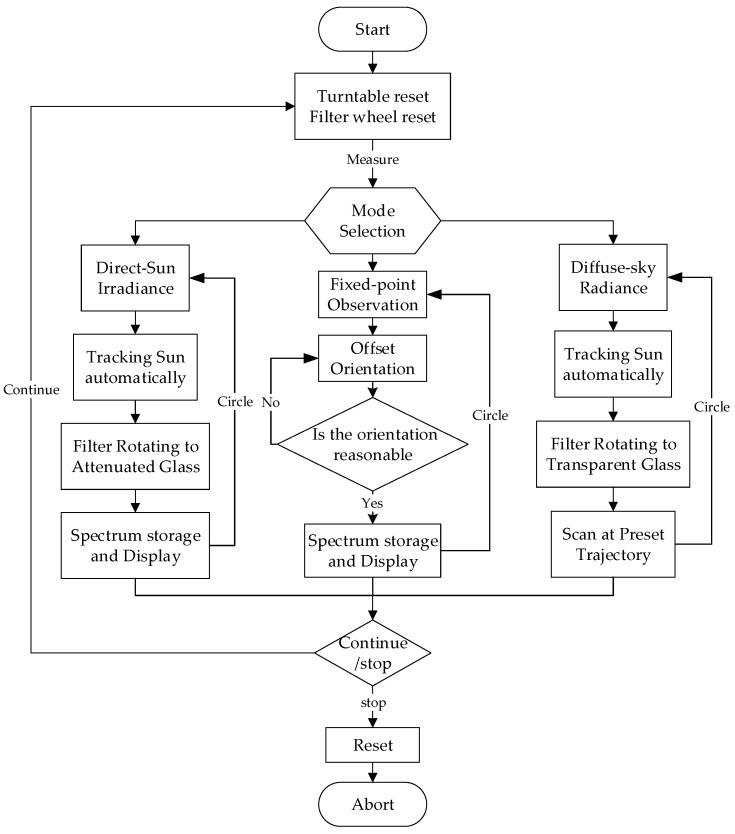
Control algorithm.

The control algorithm flow of the spectroradiometer is illustrated in Figure 8.

## 3. Calibration

The spectral response of any solar-sky radiometer is subjected to drift, so a regular instrument calibration is fundamental for a spectroradiometer to obtain quantitative optical parameters and high-quality measurements. For diffuse-sky radiance measurement, the instrument is calibrated in the laboratory by using a standard integrating sphere [24]. For direct-sun irradiance measurement, the Langley plot method derived from the Beer–Lambert law is adopted in the non-absorption band of the spectrum, and the modified Langley method will determine accurate calibration coefficients in the molecular absorption band [25].

### 3.1. Calibration of Diffuse-Sky Radiance Measurement

The integrating sphere is a standard radiation source, which can output stable and varying spectral radiance. Therefore, the spectral response of the radiometer to the standard radiance of the sphere can be expressed as [26]:

(1) $$R_{DN}=A_{0}(\lambda )+A_{1}(\lambda )L_{1}(\lambda )+A_{2}(\lambda )L_{2}{}^{2}(\lambda )+A_{3}(\lambda )L_{3}{}^{3}(\lambda )+\ldots +A_{n}(\lambda )L_{n}{}^{n}(\lambda )$$

where $L_{1}(\lambda )$ is the monochromatic radiance at wavelength λ, $R_{DN}$ is the response value of the spectroradiometer to standard input, $A_{0}(\lambda )$ is the dark noise, and $A_{1}(\lambda )$, $A_{2}(\lambda )$ are the calibration coefficients to be determined. Equation (1) can be fitted to the linear function for the case where the high-order coefficients are neglected:

(2) $$A_{1}(\lambda )=\frac{R_{DN}-A_{0}(\lambda )}{L_{1}(\lambda )}$$

The integral radiance $L_{\lambda_{1},\lambda_{2}}$ between $\left[\lambda_{1},\lambda_{2}\right]$ is given by:

(3) $$L_{\lambda_{1},\lambda_{2}}=\int_{\lambda_{1}}^{\lambda_{2}}L_{1}\left(\lambda \right)d\lambda$$

### 3.2. Calibration of Direct-Sun Irradiance Measurement

As a calibration technology in passive remote sensing, Langley extrapolation will achieve high calibration accuracy once the test site satisfies clear and stable atmospheric conditions [27]. As the radiometer output voltage V(λ,t) is linearly related to direct input radiant energy F(λ,t), the basic formula (4) of direct solar irradiance that passes through the Earth’s atmosphere can be fitted to a linear equation (Equation (5)) with slope τ(λ,t):

(4) $$F\left(\lambda ,t\right)=F_{0}\left(\lambda ,t\right)\times {\left(\frac{d}{d_{0}}\right)}^{2}\exp\left[-m\tau \left(\lambda ,t\right)\right]$$

(5) $$\ln\left[V\left(\lambda ,t\right)/{\left(\frac{d_{0}}{d}\right)}^{2}\right]=\ln V_{0}\left(\lambda ,t\right)-m\tau \left(\lambda ,t\right)$$

- V(λ,t), $V_{0}\left(\lambda ,t\right)$ is the output voltage produced by the spectroradiometer at wavelength λ when points to the sun at the ground and at the atmospheric top, respectively. $d/d_{0}$ is the relative Earth–Sun correction factor.
- m is the optical airmass, which describes the increase in the direct optical pathlength from the sun to the detector.
- $\tau_{\lambda }$ is the total atmospheric optical depth at wavelength λ, equal to the sum of aerosol ($\tau_{a}$), ozone ($\tau_{m}$) and Rayleigh ($\tau_{r}$) optical depth.

During the Langley calibration, $\tau_{\lambda }$ can be regarded as a constant once the atmosphere is clear and stable. In the Langley plot, $\ln V_{0}\left(\lambda ,t\right)$, determined by extrapolating the linear curve to the top of the atmosphere (air mass = 0), is the calibration constant used at different wavelengths. As for the water vapor absorption band, aerosol optical depth (AOD) at the absorption band can be calculated by linear interpolation from AOD at two non-absorption bands, after which the modified Langley is performed to calculate the calibration coefficients of the water vapor band.

**Figure 9:**
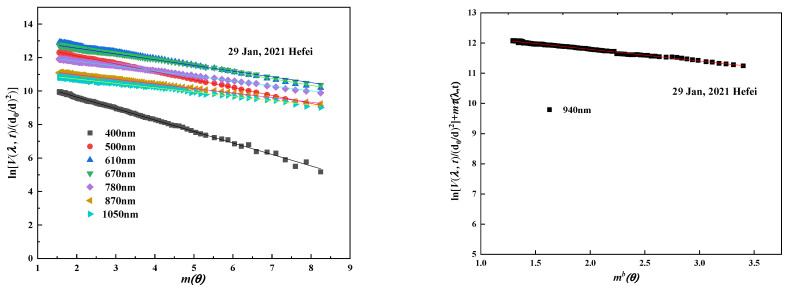
Illustration of linear relation between log radiometer voltage and optical airmass.

Although the spectroradiometer can obtain spectrum calibration coefficients in the wideband, the DTF sun-photometer acting as the reference is limited to several discrete wavelengths. The DTF sun-photometers are made up of eight channels (400 nm, 500 nm, 610 nm, 670 nm, 780 nm, 870 nm, 940 nm, 1050 nm). Therefore, only eight bands with the same central wavelength as the DTF sun-photometer are listed. The Langley plot at selected wavelengths for measurements is shown in Figure 9:

Calibration value In*G*_0_, correlation coefficient R, and standard deviation SD of eight selected bands are shown in Table 2. The correlation coefficient of each waveband is above 0.995, and the standard deviation is lower than 0.003. This indicates that least-squares fitting of the line through the data points on 29 January 2021 yields ideal results.

## 4. Result Analysis

### 4.1. Verification of Transmittance and Water Vapor

Atmospheric transmittance and total column water vapor (TCWV) are basic parameters that reflect the properties of atmospheric radiative transfer. Detailed studies on water vapor and transmittance are required in remote sensing of the Earth, climate change research and air quality monitoring. In the non-absorption waveband, the solar radiometer can calculate the atmospheric transmittance based on the ratio of measured direct irradiance to extraterrestrial solar radiation. In addition, the 940 nm water vapor band can be used to accurately retrieve TCWV [28].

#### 4.1.1. Whole Atmospheric Transmittance

In order to verify the accuracy of the spectroradiometer in measuring atmospheric transmittance, we have carried out simultaneous observation experiments between the spectroradiometer and the DTF sun-photometer in Hefei, China. Because the full bandwidth at half maximum of the two instruments is different, it is necessary to integrate the data of the spectroradiometer at the same center wavelength and bandwidth with DTF.

**Figure 10:**
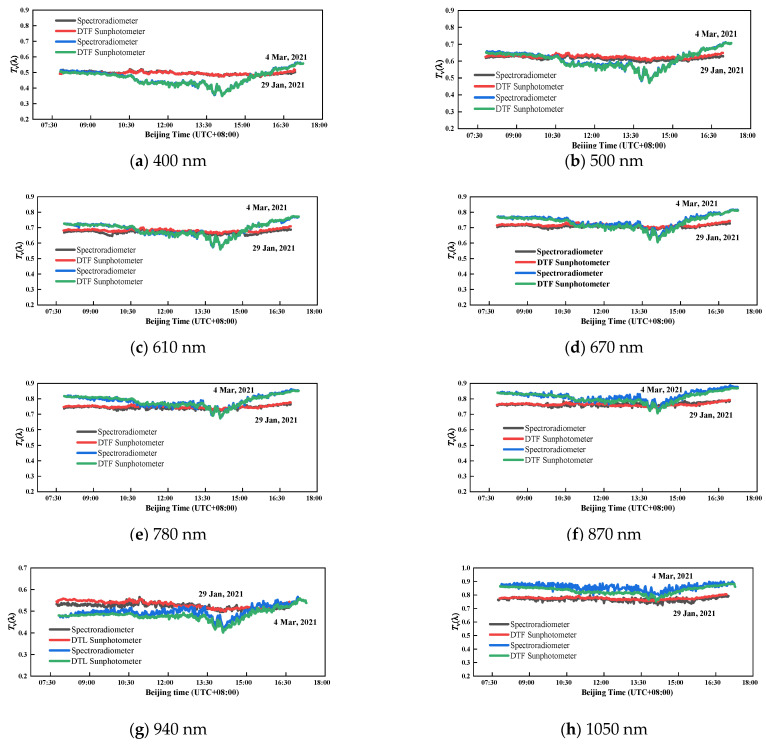
Comparison of atmospheric transmittance between spectroradiometer and DTF sun-photometer in the eight selected bands.

The atmospheric transmittance of eight typical wavelengths throughout the day is shown in Figure 10. The relative measurement differences are within 5% and 8% at each band on 29 January and 4 March, respectively, when compared to the results of the DTF sun-photometer. In particular, the transmittance in the visible region of the two instruments is consistent, which reflects the variation of the direct solar irradiance in radiative transfer. The experiments show that the performance of spectroradiometer is relatively accurate and stable.

#### 4.1.2. Total Water Vapor

Under clear weather conditions, the solar radiometer calculates the AOD of the water vapor band based on the Ångström formula or AOD linear interpolation method; then, the water vapor content can be obtained by the inversion model [29]. The microwave radiometer (MP-3220A) is a highly sensitive instrument for measuring the atmospheric water vapor profile using multi-band microwave radiation [30]. The microwave can penetrate cloud and atmosphere, and cover more than 10 km of the atmosphere. The measurement results of MP-3220A can approximately represent the total water vapor in the whole atmosphere.

**Figure 11:**
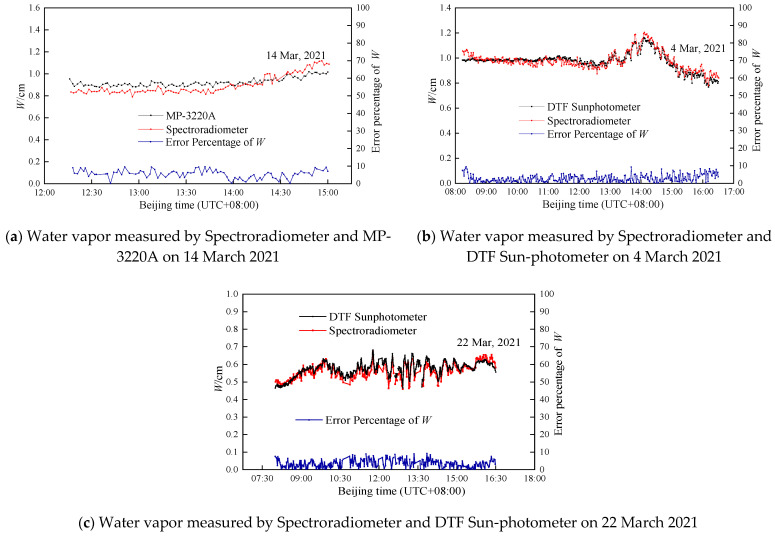
Graphs of the water vapor column of the spectroradiometer, DTF sun-photometer and microwave radiometer, respectively.

Although the water vapor results of the spectroradiometer and reference are in good agreement, there are still relative deviations, as shown in Figure 11. It is important to point out that the spectroradiometer, the DTF sun-photometer and the microwave radiometer use three different detectors. The error source of the measurement may come from the difference in response characteristics of the detectors, the calibration error of the instruments, and the influence of the absorption gas.

### 4.2. Continuous Spectrum Transmittance

The attenuation process of molecular absorption and scattering of solar radiation in transmission contains abundant atmospheric molecular information and optical properties. In order to accurately analyze the atmospheric radiation based on the Earth’s surface radiation, it is necessary to measure and study the continuous spectrum transmittance of the featured spectral band, and understand the variation characteristics of the spectral transmittance under the actual atmospheric model.

**Figure 12:**
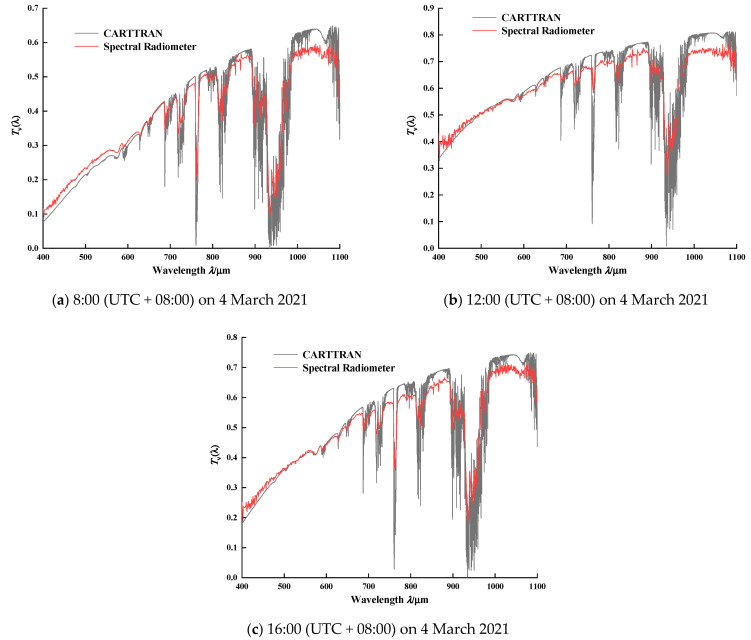
The continuous spectrum transmittance between spectroradiometer and CART in the morning, noon, and afternoon on 4 March 2021. Note the agreement of these observations between the two methods.

Combined Atmospheric Radiative Transfer (CART) can quickly calculate the spectral transmittance and atmospheric scattering characteristics, with spectral resolution of 1 cm^−1^ and a spectral range of 1~25,000 cm^−1^ [31]. Figure 12 illustrates the comparison of spectral transmittance between the CART and spectroradiometer in the morning, noon, and afternoon. The transmittance curve of the spectroradiometer can characterize the absorption position of water vapor, oxygen, ozone and other molecules. Moreover, the variation trend in 400~1100 nm of the spectroradiometer is in good conformity with that of CART, and the relative depth of the water vapor absorption valley is approximately the same. The comparison results verify the accuracy of the instrument in obtaining continuous spectrum transmittance. The relative difference is mainly due to the following uncertainties: In the process of CART simulation, the input parameters are not completely consistent with the actual atmospheric conditions. In addition, the spectroradiometer does not take into account the influence of aerosols, cirrus clouds and other factors when analyzing the transmittance, which also leads to the relative error.

**Figure 13:**
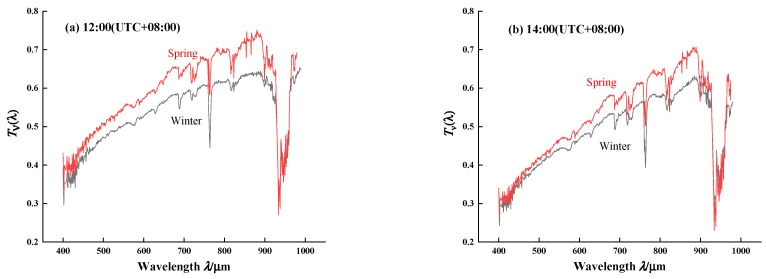
Comparison of continuous spectrum transmittance in winter and spring.

Figure 13 shows comparison result of spectral transmittance between winter (January) and spring (March). Even though the decline of atmospheric altitude in winter leads to a decrease in molecules absorption, the spectral transmittance in winter is significantly lower compared to that in spring, especially in the shortwave region. However, in the red region, such as the water vapor absorption band, the spectral transmittance tends to coincide. Due to the fact that the experimental site, Hefei, is located north of the equator, the solar zenith angle in winter is obviously greater than that in spring. With the increase in solar zenith angle and the Earth–Sun distance, the spectral energy will be absorbed and scattered by more atmospheric molecules. Therefore, the spectral atmospheric transmittance measured in winter is lower compared to that observed in spring, especially the shortwave radiation that is easily scattered by molecules.

**Figure 14:**
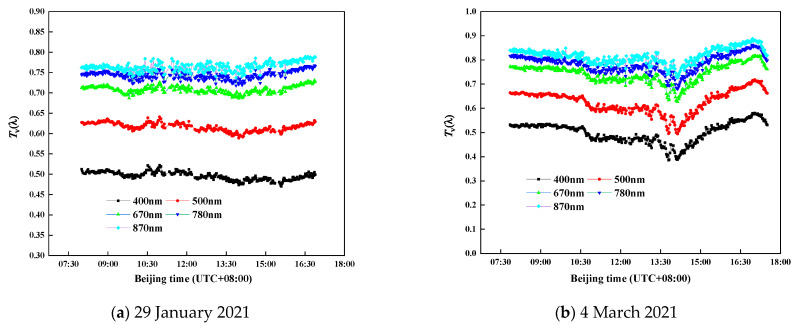
Atmospheric transmittance of five typical wavelengths during the course of a day.

Figure 14 illustrates the atmospheric vertical transmittance at five wavelengths of 400 nm, 500 nm, 670 nm, 780 nm, and 870 nm during the two observation days on 29 January and 4 March, respectively. With increasing wavelengths, the value of the entire atmospheric transmittance of four wavelengths increases gradually. The corresponding continuous spectrum transmittance of wavelengths in Figure 12 also shows an increasing trend from visible to near-IR, which further verifies the reliability of the measured result.

### 4.3. Diffuse-Sky Radiance

In the diffuse-sky radiance observation, the spectroradiometer provides a featured angular sky scanning, and the sky hemisphere is divided into 354 grids. The measuring probe can rotate in two orthogonal dimensions of pitch and level, so as to obtain the absolute integrated radiance in the whole sky. Accurate measurement of sky radiation will contribute toward improving the accuracy of space imaging. Furthermore, the optical thickness and effective radius of clouds can be determined from solar radiation measurements [32,33].

**Figure 15:**
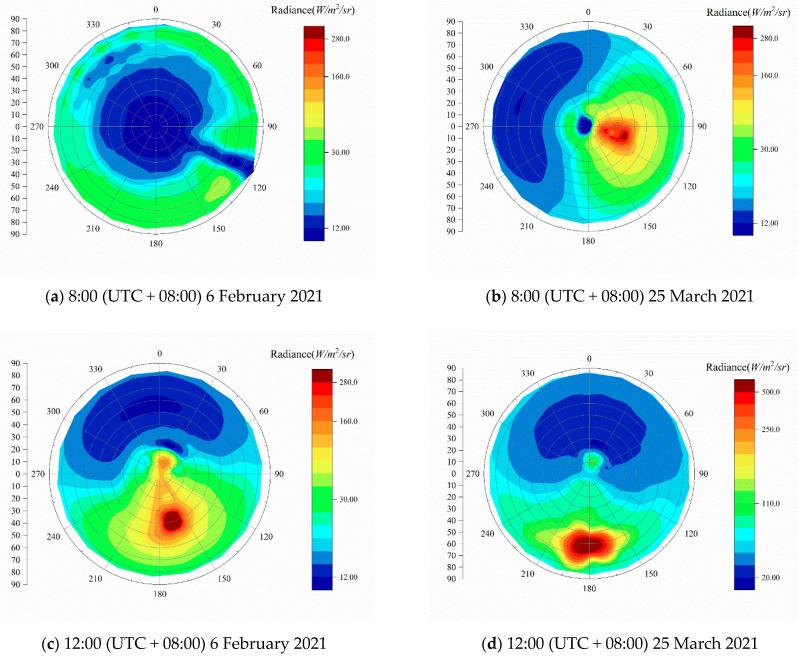

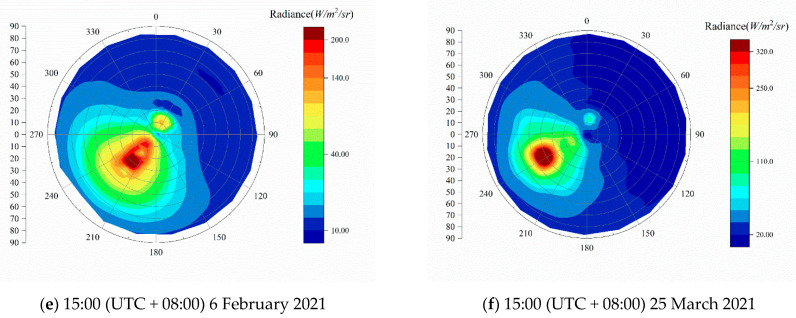
Diffuse-sky radiance distribution at different times during the course of a day.

Figure 15 shows three snapshots of the diffuse-sky radiance information throughout the day on February 6 and March 25, respectively. The absolute integrated radiance on March 25 is significantly higher compared to that on February 6 at the same time. This difference is mainly due to the change in solar zenith angle in the observation area from winter to summer. Moreover, the diffuse-sky radiance distributions of the two days have some similarities:

- The radiance distribution under cloud conditions (Figure 15a) is affected by the clouds and the solar zenith angle, which presents an ambiguous result and no obvious regularity.
- On a sunny day, the radiation in the whole sky shows a symmetrical distribution concerning the line connecting the Sun and the zenith. The solar zenith angle is the main factor determining the value of sky radiance. With the increase in solar zenith angle, the sky radiance decreases accordingly.

**Figure 16:**
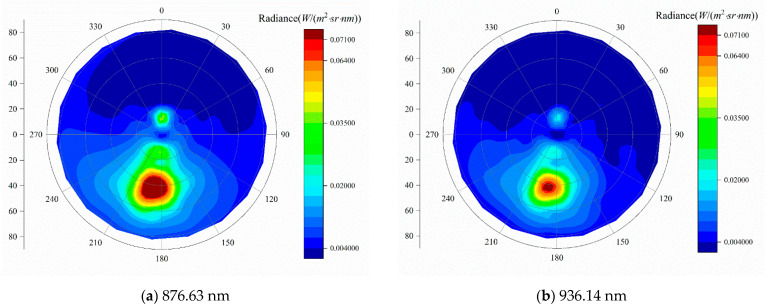
Two images of sky radiation distribution at single wavelength.

Two images of sky radiance at the single wavelength are illustrated in Figure 16. At the same observation time, the diffuse-sky radiance distributions at different wavelengths have some similarities, but there are various areas with highlighted regions in the distribution graph. The single-wavelength background radiance on the absorption band is significantly smaller than that of its adjacent non-absorption band. For example, the radiance value of 936.14 nm in the water vapor absorption band is an order of magnitude smaller than that of 876.63 nm.

### 4.4. Fixed-Point Radiance

**Figure 17:**
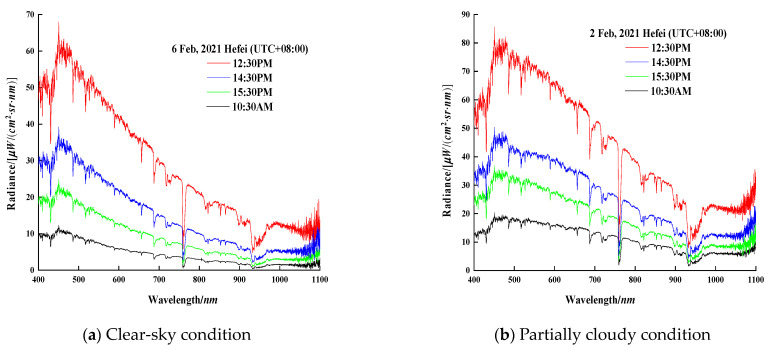
Fixed-point spectral radiance for two days in Hefei.

For the fixed-point radiance observation, the measuring probe of the spectroradiometer points to a given azimuth (180°, 30°) in the sky for fixed-point and equal-period observations. Figure 17a shows fixed-point radiation under a clear sky on 6 February; Figure 17b presents the measurement results under partially cloudy on 2 February:

- Under clear-sky conditions, the radiation curve is relatively smooth with slight fluctuation. With the solar zenith angle becoming lower, the proportion of shortwave radiation decreases and the long-wave radiation increases correspondingly.
- The existence of clouds will affect the distributions of sky radiation. In a partially cloudy condition, the radiation distributions in the entire waveband are relatively uniform, and the curve descent tends to be gentle. The proportion of long-wave radiation is significantly higher when compared to that in the clear-sky condition.

## 5. Conclusions

This paper proposes a scheme for developing a solar-skylight spectroradiometer with wideband observation of 380~1100 nm. Through the deep integration between hardware and software resources, the embedded Linux integrated with the communication protocol family can control sensor networks and perform data analysis. (1) In terms of direct solar irradiance measurement, the whole atmospheric transmittance of the spectroradiometer shows the maximum relative difference of 8% when compared to the DTF sun-photometer. The relative error of total water vapor is within 10% compared to that of the microwave radiometer. (2) The consistency of the continuous spectrum transmittance between the spectroradiometer and the CART software confirms the effectiveness of the measurement system. (3) For the diffuse-sky radiance, in contrast to a traditional radiometer which measures discrete wavebands on a preset orbit, a spectroradiometer can observe the radiation distributions and spectral information of the whole sky. The implementation of measurement technology can enrich the methods to obtain atmospheric parameters such as direct solar irradiance, sky radiance and total solar radiation, and provide refined spectral distributions information.

## Figures and Tables

**Table 1:** Main features of spectroradiometer.

Performance Characteristics	Specifications
Measurement waveband	380~1100 nm
Spectrum resolution	<1 nm
Measuring mode	direct-sun irradiance, diffuse-sky radiance
Field of view	0.8°
Tracking angle resolution	<5 arcsec
Positioning accuracy	<40 arcsec
Control architecture	ARM-Linux architecture
Working temperature	−30~55 °C

**Table 2:** Calibration value, correlation coefficient and standard deviation of eight bands.

Date	Parameter	Selected Eight Wavebands							
		400 nm	500 nm	610 nm	670 nm	780 nm	870 nm	940 nm	1050 nm
29 January 2021	In*G*_0_	11.0146	13.0431	13.5085	13.2467	12.3778	11.52	12.5569	11.236
	R	−0.9990	−0.9994	−0.9986	−0.9988	−0.9985	−0.9984	−0.9957	−0.9969
	SD	0.00237	0.00132	0.00163	0.00132	0.00126	0.0012	0.00261	0.00163
